# A Novel and Lethal *De Novo* LQT-3 Mutation in a Newborn with Distinct Molecular Pharmacology and Therapeutic Response

**DOI:** 10.1371/journal.pone.0001258

**Published:** 2007-12-05

**Authors:** John R. Bankston, Minerva Yue, Wendy Chung, Meghan Spyres, Robert H. Pass, Eric Silver, Kevin J. Sampson, Robert S. Kass

**Affiliations:** 1 Department of Pharmacology, College of Physicians and Surgeons, Columbia University Medical Center, New York, New York, United States of America; 2 Department of Pediatrics, College of Physicians and Surgeons, Columbia University Medical Center, New York, New York, United States of America; 3 College of Physicians and Surgeons, Columbia University Medical Center, New York, New York, United States of America; University of Cincinnati, United States of America

## Abstract

**Background:**

*SCN5A* encodes the α-subunit (Na_v_1.5) of the principle Na^+^ channel in the human heart. Genetic lesions in *SCN5A* can cause congenital long QT syndrome (LQTS) variant 3 (LQT-3) in adults by disrupting inactivation of the Na_v_1.5 channel. Pharmacological targeting of mutation-altered Na^+^ channels has proven promising in developing a gene-specific therapeutic strategy to manage specifically this LQTS variant. *SCN5A* mutations that cause similar channel dysfunction may also contribute to sudden infant death syndrome (SIDS) and other arrhythmias in newborns, but the prevalence, impact, and therapeutic management of *SCN5A* mutations may be distinct in infants compared with adults.

**Methods and Results:**

Here, in a multidisciplinary approach, we report a *de novo SCN5A* mutation (F1473C) discovered in a newborn presenting with extreme QT prolongation and differential responses to the Na^+^ channel blockers flecainide and mexiletine. Our goal was to determine the Na^+^ channel phenotype caused by this severe mutation and to determine whether distinct effects of different Na^+^ channel blockers on mutant channel activity provide a mechanistic understanding of the distinct therapeutic responsiveness of the mutation carrier. Sequence analysis of the proband revealed the novel missense *SCN5A* mutation (F1473C) and a common variant in *KCNH2* (K897T). Patch clamp analysis of HEK 293 cells transiently transfected with wild-type or mutant Na^+^ channels revealed significant changes in channel biophysics, all contributing to the proband's phenotype as predicted by *in silico* modeling. Furthermore, subtle differences in drug action were detected in correcting mutant channel activity that, together with both the known genetic background and age of the patient, contribute to the distinct therapeutic responses observed clinically.

**Significance:**

The results of our study provide further evidence of the grave vulnerability of newborns to Na^+^ channel defects and suggest that both genetic background and age are particularly important in developing a mutation-specific therapeutic personalized approach to manage disorders in the young.

## Introduction

The long QT syndrome (LQTS), a relatively uncommon (1 in 2500) genetic disorder associated with life-threatening arrhythmias, is an example of a channelopathy that has provided a wealth of information about fundamental mechanisms underlying human cardiac electrophysiology[1]. LQTS is currently associated with mutations in 10 different genes[1]–[3]. LQT variant 3 (LQT-3) is caused by mutations in *SCN5*A, the gene coding for the alpha subunit (Na_V_1.5) of the primary cardiac voltage-gated Na^+^ channel in the human heart. Despite a relatively common phenotype (delayed repolarization reflected in QT prolongation), therapeutic strategies have been developed that are dictated by molecular etiology. Na^+^ channel blockers such as mexiletine and flecainide are effective in treating LQT-3 patients due to preferential inhibition of mutant Na^+^ channel activity[4], [5]. Furthermore, current evidence suggests that mutations in ion channels may also contribute to sudden infant death syndrome (SIDS) and other cardiac arrhythmias in newborns, and that the frequency, severity, and treatment of *SCN5A* mutations may be distinct in infants compared with adults[6], [7], [8]–[11]

Here we report investigation of a *de novo SCN5A* mutation discovered in a newborn who had presented with extreme QT prolongation (approximately 800 ms); 2:1 heart block; and differential responses to the Na^+^ channel blockers lidocaine, mexiletine, and flecainide. Also found in the proband was common polymorphism (K897T) in *KCNH2,* the gene coding for the HERG potassium channel which contributes to control of ventricular repolarization. Our results reveal multiple biophysical changes in Na^+^ channel gating induced by the F1473C mutation that all contribute to delayed repolarization of cellular action potentials and are consistent with the pronounced phenotype of the patient. Furthermore our data suggest an important role of the *KCNH2* polymorphism and developmentally-related expression of background ion channels in both the therapeutic response and disease phenotype of the proband.

## Results

### Clinical profile

**Figure 1:**
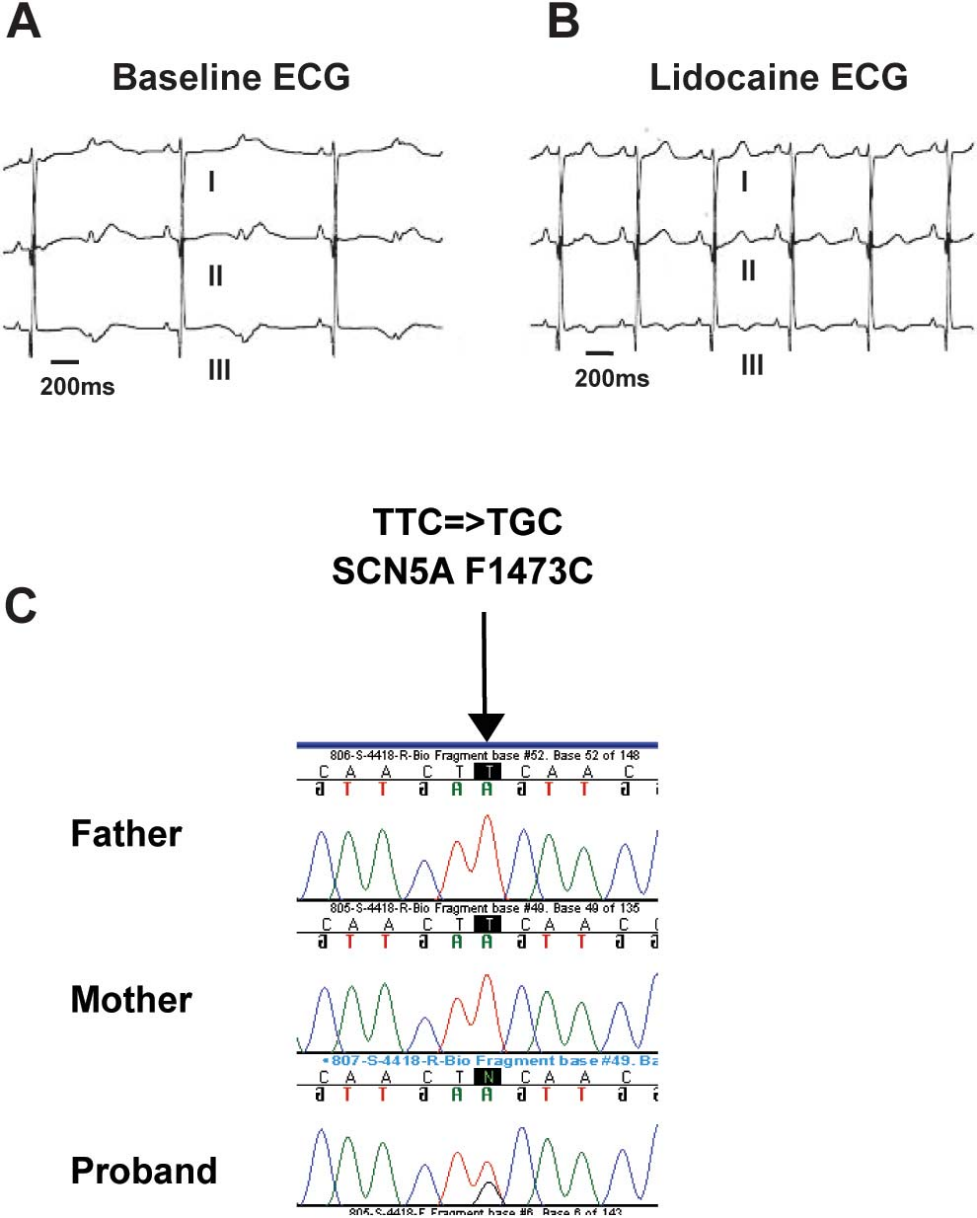
Clinical and genetic profile of the proband. A. Baseline ECG recording reporting a QTc of approximately 825 ms. Note 2∶1 AV block. B. Recording after infusion with IV lidocaine (see text). QTc is approximately 450 and 2∶1 block is absent. C. Chromatograms of DNA sequences: the proband but neither the mother nor father has a 4418 T = >G in *SCN5A* resulting in a substitution of cytsteine for phenylalanine at amino acid 1473 in Na_v_1.5.

The proband was a full term newborn infant boy with a prenatal history significant for fetal bradycardia of unclear etiology with a normal structural heart. There was no family history of arrhythmias, long QT syndrome, or sudden death. His ECG demonstrated 2:1 AV block (atrial rate of 108 and ventricular rate of 54) with a corrected QT_C_ of roughly 825 ms (Fig 1A). Multiple short episodes of torsades de pointes were documented and treated initially with IV magnesium and esmolol. Because review of the ECG suggested a possible *SCN5A* mutation based upon the morphology of the QT interval, the patient was given a test dose of IV lidocaine (1 mg/kg) which resulted in a profound drop in the corrected QT interval to less than 460ms within 15 seconds of the bolus dose (Fig. 1B). Approximately 30–45 minutes after this dose, the QT interval lengthened to roughly 550 ms with intermittent 2∶1 conduction. In an attempt to convert the patient to oral Na^+^ channel blockers, flecainide was administered with no appreciable change in the QT interval and with continued incidences of torsades. Mexilitene was ineffective at lower doses (1–3 mg/kg/dose po q 6 hours), but at higher doses (∼6 mg/kg/dose) shortened the QT interval (∼550–600 ms) similarly to IV lidocaine. However, the patient continued to have 1–5 short episodes of torsades each day which led to a dual chamber implantable cardioverter defibrillator/pacemaker being implanted epicardially, and it was observed that with ventricular pacing (dual mode pacing with a lower rate limit of 125/minute) there was marked reduction in episodes of torsades. On a regimen of oral beta blockade at high dose (∼4–5 mg/kg/day of propranolol/inderal) with high dose mexiletine, oral magnesium supplementation and AV sequential pacing, the infant was ultimately discharged from the hospital at approximately 3 months of age. There was no family history of prolonged QT, syncope, presyncope, or sudden death. Both parents had screening ECGs with normal QT intervals. This was the parents' only child. The family was of Italian and Portugese ancestory.

### Genetics

Sequence analysis of the proband identified a 4418T = >G *SCN5A* variant resulting in a F1473C missense mutation in Na_v_1.5 (Fig 1C). In addition, the proband was heterozygous for the 2690 A = >C *KCNH2* polymorphism producing a missense K897T variant in the HERG channel protein. No mutations were identified in *KCNQ1* (codes for the α subunit of the slowly-activating I_KS_ channel), *KCNE1*(codes for the β subunit of the I_KS_ potassium channel*)*, or *KCNE2* (codes for the β subunit of the rectifying I_Kr _potasium channel) or other channel-related LQTS genes. Neither parent was found to have the F1473C mutation. The father was homozygous for the K897 *KCNH2*, and the mother was homozygous for the T897 *KCNH2* polymorphism. Microsatellite markers confirmed maternity and paternity and establish F1473C *SCN5A* as a de novo mutation. None of the 100 Caucasian controls were found to carry the F1473C mutation.

### Biophysical Consequences of the F1473C mutation: multiple mechanisms contribute to delayed repolarization

**Figure 2:**
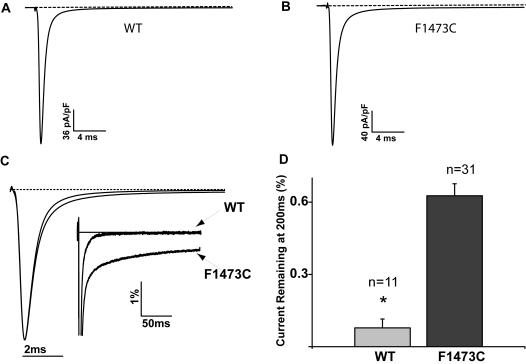
The F1473C mutation increases non-inactivating late current. Whole-cell wild type (WT, A) and mutant (F1473C, B) Na^+^ channel currents at −10 mV (200 ms pulses applied from a −100 mV holding potential at 0.5 Hz). Shown are averaged raw TTX sensitive traces (WT, n = 11; F1743C, n = 31). C. Averaged WT and F1473C currents normalized to peak current and superimposed at high (left) and low (inset) gain (WT n = 11, F1473C n = 31). D. Bar graph representing mean +/− S.E.M. percentage of current remaining at 200 ms with respect to peak current for both WT and F1473C (WT n = 11, F1473C n = 31); * p<<0.001.

The severity of the clinical phenotype, the differential response to pharmacological agents, and the location of this mutation within the Na_v_1.5 inactivation gate (DIII_DIV linker), made the biophysical consequences of the F1473C mutation of particular interest to us. Figure 2 summarizes the first set of parameters we examined using standard whole cell patch clamp recording procedures and results of experiments that were designed to focus on potential effects of the mutation on currents recorded during prolonged depolarization. These currents will be referred to as late Na^+^ channel currents. Figure 2A and B compare averaged raw TTX-sensitive current traces recorded in HEK 293 cells expressing WT (A) and F1473C mutant (B) Na^+^ channels at sufficiently low gain to capture peak Na^+^ channel currents. In order to detect potential mutation-induced physiologically-significant changes in Na^+^ channel activity during prolonged depolarization it is necessary to visualize currents at high recoding gains, and to compare putative mutation-induced channel dysfunction to other disease-related mutations, it is useful to display currents normalized to peak Na^+^ channel currents. This analysis is presented Figure 2C which shows low (left traces) and high (inset) gain recordings of superimposed averaged and normalized TTX sensitive current traces recorded at −10 mV in cells expressing WT and F1473C (arrow) channels. The impact of the mutation on channel activity is most apparent in the high gain recordings, which reveal a marked increase in Na^+^ channel activity that has failed to inactivate completely over the duration of the 200 ms test pulse. Figure 2D, which presents summary data, shows that the F1473C mutation causes a 6-fold increase in non-inactivating late current (I_NaL_) measured at 200ms during pulses to −10 mV (WT: I_NaL_ = 0.1+/−0.01%, n = 11 and F1473C: I_NaL_ = 0.63+/−0.05%, n = 31; p<1.5e-05), which is plotted as the percentage of peak current.

**Figure 3:**
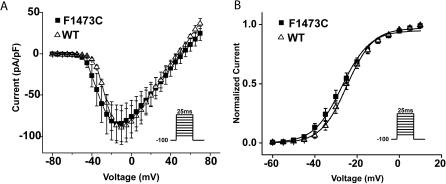
The F1473C mutation does not affect the voltage dependence of Na^+^ channel activation. A. Mean current voltage relationships recorded in low external sodium solution (Methods S1) in response to 25 ms voltage pulses from −80 mV to +75 mV (5 mV increments) for WT (n = 13, open triangles) and F1473C (n = 7, closed squares). B. Mean activation curves obtained from data in A, for WT (open) and F1473C channels (filled) show no affect of the F1473C mutation on the voltage dependence of activation (see text for details).

Figure 3 summarizes experiments focusing on possible effects of the F1473C mutation on the voltage-dependence of activation and peak current density. Because these experiments focused on peak and not late currents, we used extracellular solutions containing reduced [Na^+^] (Methods S1 (supplemental methods)) to ensure adequate voltage control. Figure 3A, which presents mean peak current voltage-relationships for WT (open triangles) and F1473C (filled squares) channels, indicates no mutation-induced change in mean current density. Analysis of these current voltage relationships reveals no detectable change in the voltage-dependence of activation (Figures 3B; WT: V_1/2_ = −24.8 +/−1.3 mV, n = 13 and F1473C: V_1/2_ = −27.2+/−1.6 mV, n = 7), slope factor of the fitted Boltzmann relationships (WT: k = 6.2+/−0.3 and F1473C: k = 6.6+/−0.2). Thus the primary functional consequence of the F1473C mutation appears to be disruption of Na^+^ channel inactivation during prolonged depolarization such as depolarization in ventricular cells during the plateau phase of the ventricular action potential underlying the QT interval of the ECG.

**Figure 4:**
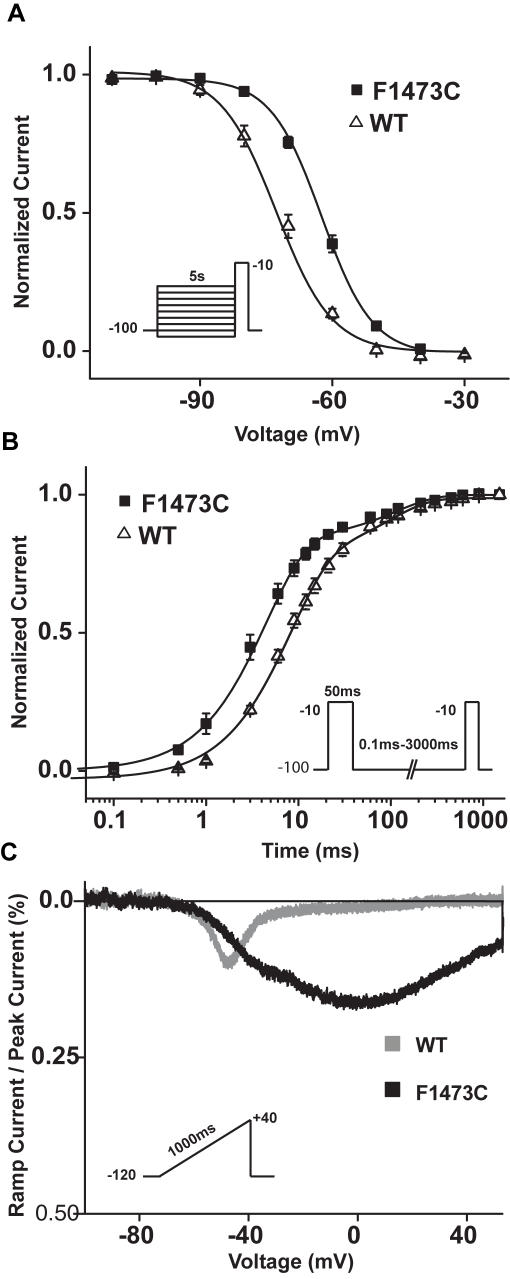
The F1473C mutation has multiple biophysical consequences. A. The F1473C mutation causes an 8.8±1.6 mV depolarizing shift in the steady state availability curve. Data shown for WT (n = 9, open triangles) and F1473C (filled squares, n = 25) channels. B. Mutation at F1473C speeds the time course of recovery from inactivation as measured at −100 mV following a 50 ms pulse to −10 mV. WT (open triangles, n = 5) and F1473C (closed squares, n = 5). C. Normalized WT (grey) and F1473C (black) currents in response to a ramp in voltage from −120 mV to +40 mV (see Methods S1) over the course of 1s reveal F1473C-enchanced inward current over a broad range of voltage. Shown are averaged TTX-sensitive currents for WT (light grey, n = 4) and F1473C (black, n = 11) channels. Note the inverted bell-shaped response of WT channels to this protocol reveals “window” of current for WT channels (see text for details).

The marked increase in I_NaL_ caused by the mutation is consistent with mutant Na^+^ channel activity that can contribute to delayed repolarization of cellular action potentials[12], but this is not the only biophysical property altered by the mutation that might contribute to the disease phenotype. Because mutation-altered Na^+^ channel inactivation is critical to the functional consequences of the mutant channels on cellular electrical activity, we next tested for effects of the F1473C mutation on steady state inactivation which primarily reflects transitions from closed to inactivated states (closed state inactivation), as well as the impact of the mutation on the time course of recovery from inactivation at negative potentials. We measured availability curves for both WT and mutant channels using conditioning pulses of 0.5 s (not illustrated) and 5 s (Figure 4A) using voltages that ranged from −120 mV to −20 mV. As shown in the figure, the F1473C mutation causes almost a +10mV depolarizing shift in the V_1/2 _of channel availability (WT: V_1/2_ = −71.2+/−0.9 mV, n = 9 and F1473C: V_1/2_ = −62.4+/−0.7 mV, n = 25; p<1.79E-07) with little or no change in the slope factor of the fitted Boltzmann relationships (WT: k = 6.4+/−0.3 and F1473C: k = 5.7+/−0.2). We illustrated the results of prolonged conditioning pulses to ensure steady state was reached for the pharmacological experiments that follow, but similar results were obtained using conditioning pulses of 500 ms (data not shown).

The time course of recovery from inactivation was investigated using a 50ms conditioning pulse (−10 mV) to induce inactivation followed by a test pulses after returning to −100 mV for varying time periods to allow time for channels to transition out of the inactivated state. The data from these experiments, fitted by functions with two exponential components, are summarized in Figure 4B. The data show that the F1473C mutation causes a significant speeding of both the fast (WT: τ_fast_ = 8.4+/−0.8 ms, n = 5 and F1473C: τ_fast_ = 4.5+/−0.6 ms, n = 5; p<0.003) and slow (WT: τ_slow_ = 216.74+/−39.3 ms n = 5 and F1473C: τ_slow_ = 112.6+/−19.8 ms n = 5; p<0.04) processes by which Na^+^ channels transition from inactivated to available (closed) sates. The results summarized in Figures 2 and 4 show that the inherited mutation makes channels reluctant to enter the inactivated state (Figures 2C,D and 4A), and shortens the time in the inactivated state once channels are returned to voltages in the range of diastolic potentials (Figure 4B). Interestingly, similar results have been reported in experiments with recombinant skeletal muscle F1473C Na^+^ channels[13]. Furthermore, although the mutation-induced positive shift in steady-state inactivation (Figure 4A) but not activation (Figure 3B) slightly-changes the voltage-dependence of Na^+^ channel window current, the mutation clearly increases late currents measured over a broad ranges of voltages (Figure 4C). In the experiments illustrated in Figure 4C, positive voltage ramps (Methods) were imposed on HEK 293 cells expressing WT and F1473C channels (see labels in figure), in the absence and presence of TTX. Shown in the figure are TTX-sensitive currents for both conditions. In response to positive voltage WT channels conduct current over a narrow range of voltages (peaking near −40 mV in these experiments). This type of current is referred to as “window” current due to overlap between activation and inactivation relationships in this narrow voltage range. Clearly F1743C mutant channels conduct not only over this narrow voltage range, but over the full range of voltages that open the channels. Thus altered window current by itself does not account for the F1473C-induced increase in late current.

### Distinct Pharmacology of F1743C Mutant Channels

**Figure 5:**
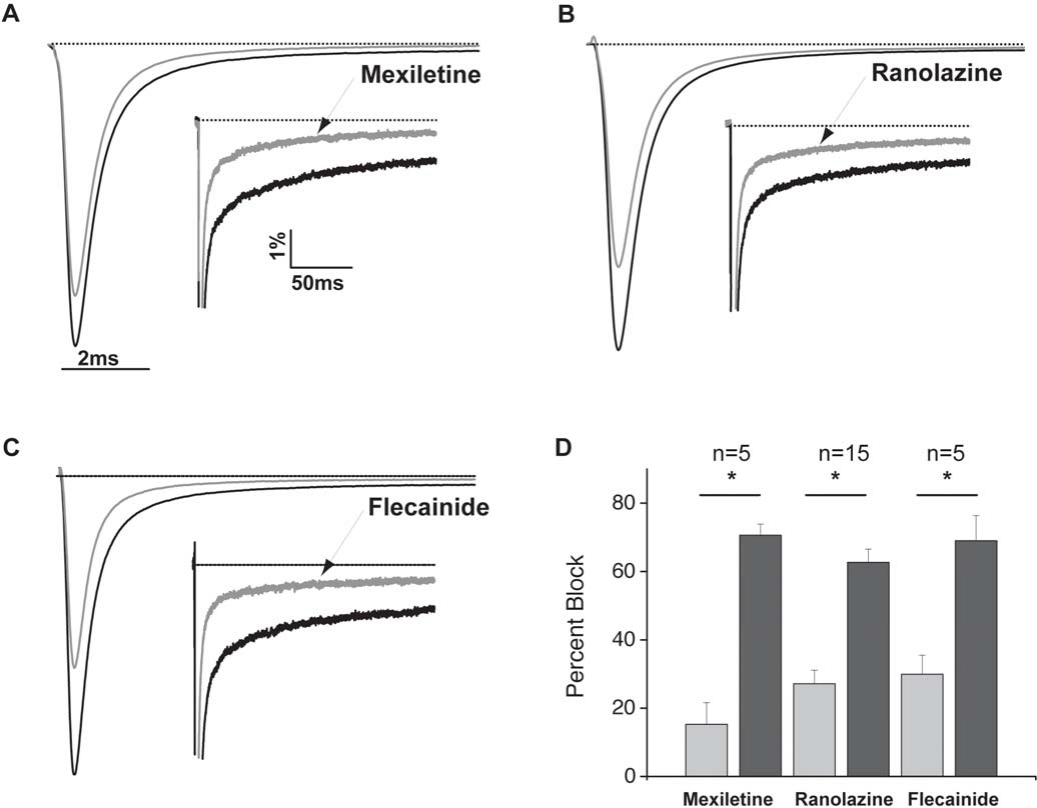
Relative sensitivity of peak and late F1473C channel current to three different Na^+^ channel blockers. A–C. Averaged normalized currents from F1473C channels evoked by 200 ms depolarizing currents to −10 mV at 0.5Hz in the presence and absence of 50 µM mexiletine (A, n = 5), 50 µM ranolazine (B, n = 15), and 10 µM flecainide (C, n = 5). Traces in the presence of each drug are shown in grey and indicated by an arrow in the inset. Drug-free traces are black. Note that the units and presentation reflect normalization to peak current under drug-free conditions. D. Bar graph summarizes fraction of peak (grey bars) and late current (black bars) blocked by each drug. Each drug inhibited late current preferentially over peak current (* p<<0.05). Inhibition of peak and late current was not significantly different among all drugs tested.

**Figure 6:**
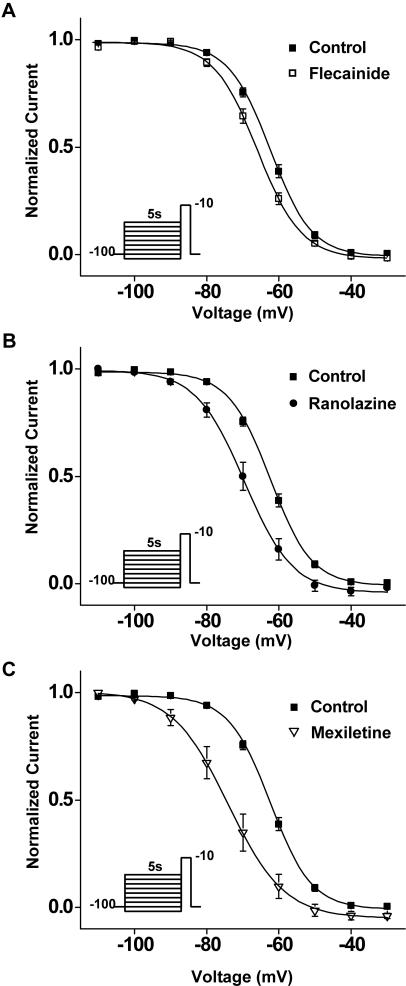
Mexiletine and Ranolaziine, but not Flecainde, correct mutation-induced voltage shift in steady state inactivation. Steady state inactivation was measured with a 5s conditioning pulse (see Methods S1) followed by a brief −10 mV test pulse. A. Flecainide (n = 4) did not significantly alter V_1/2_ or slope factor of the channel availability curve. B–C. Mexiletine (n = 5) and Ranolazine (n = 9) each caused significant hyperpolarizing shifts in the voltage-dependence of steady-state inactivation (Table 1).

The differential response of the patient to Na^+^ channel blockers prompted us to investigate the efficacy of three different compounds, flecainide, mexiletine, and ranolazine, in correcting the biophysical dysfunction of the F1473C mutant channel. We selected these drugs because, in cases of other LQT-3 mutations, each has been shown to inhibit preferentially late vs. peak Na^+^ channel currents and, at least in the case of flecainide and mexiletine, has been shown effective in treating patients carrying LQT-3 mutations[14], [15]. The three drugs (Figure 5) more potently inhibit late vs. peak F1473C channel currents, with roughly the same selectivity reported previously for channels carrying the ΔKPQ LQT-3 mutation[16], [17]. Figures 6B and 6C reveal that, at concentrations that significantly inhibit I_NaL_ (Figure 5), both ranolazine (50 µM) and mexiletine (50 µM ranolazine) cause similar and significant hyperpolarizing shifts in the voltage-dependence of steady-state inactivation for F1473C channels (Mexiletine: −11.2 mV shift, p<0.009 and Ranolazine: −6.6 mV shift, p<0.003). These shifts in inactivation, comparable for these two drugs, essentially restore the closed state inactivation voltage range of WT channels to F1473C channels (Table 1). In contrast, a flecainide concentration (10 µM) which has comparable efficacy on I_NaL_ inhibition (Figure 5), has no significant effect on the voltage-dependence of F1473C channel closed state inactivation (Figure 6A, Table 1), consistent with previous reports on the effect of flecainide on steady state inactivation for WT channels[18].

**Table 1:** Effects of Na^+^ channel blockers on WT and F1473C channels.

	Peak Current Block (%)	Late Current Block (%)	Inactivation V_1/2_ (mV)	Inactivation slope factor
WT			−71.2+/−0.9 (9)	6.4+/−0.3
WT Mexiletine	24.6+/−2.7 (5)		−85.5+/−3.2 (4) *	7.6+/−0.6
WT Ranolazine	13.2+/−3.2 (6) §		−76.1+/−1.6 (5) *	6.3+/−0.4
F1473C			−62.4+/−0.7 (25)	5.7+/−0.2
FC Flecainide	30.0+/−5.6 (5)	69.0+/−7.4 (5)	−64.3+/−1.0 (4)	6.1+/−0.5
FC Mexiletine	15.3+/−6.3 (5)	70.6+/−3.3 (5)	−73.6+/−2.6 (5) †	7.1+/−0.4‡
FC Ranolazine	27.2+/−3.9 (15) §	62.7+/−3.9 (15)	−69.0+/−1.6 (9) †	6.2+/−0.4

Table 1 summarizes the values for current block in both WT channels and F1473C channels in the presence of the drugs tested. Also shown is the average values of the Boltzmann fit parameters for the steady state availability curve. Values are reported as the mean±S.E. The number in parentheses indicates the number of experiments. Statistical significance was determined using an unpaired Student's t test.

* p<0.03 vs WT V_1/2_,

† p<0.009 vs FC V_1/2_,

‡ p<0.03 vs FC slope factor,

§ p<0.01 vs WT block in Ranolazine.

### Impact of the F1743C mutation and Na^+^channel blockers on Na^+^ channel currents during repolarization

**Figure 7:**
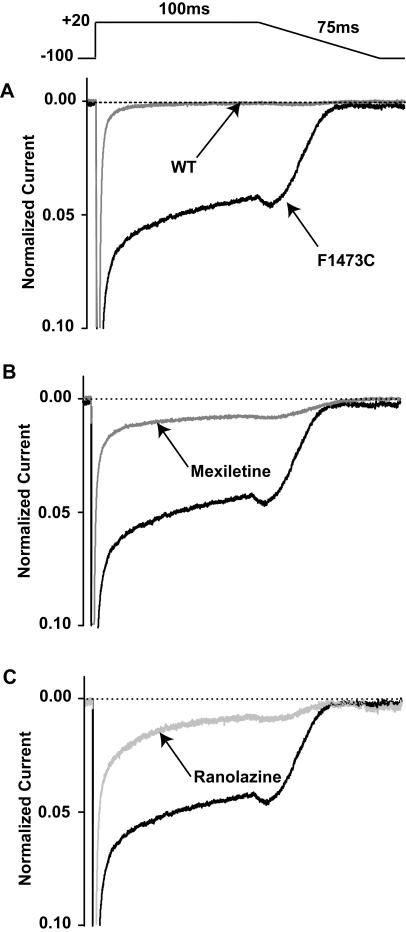
Mexiletine and ranolazine inhibit F1473C channel activity during repolarization. Inward currents were measured in response to a pulse to +20 mV for 100 ms followed by repolarization to −100 mV at −1.6 V/sec (negative ramp) (see Methods S1). A. F1473C channels, relative to WT channels, show a very large increase in non-inactivating current for the duration of the voltage pulse to +20 mV and also cause a large surge of inward current during the negative voltage ramp. B–C. Mexiletine (B) and ranolazine (C) inhibit the mutation-induced repolarizing inward current (Mexiletine block of repolarization phase peak current = 82.1+/−0.2%, n = 7; Ranolazine block of repolarization phase peak current = 83.1+/−0.2%, n = 4).

Mutations that enhance Na^+^ channel activity either through altered channel kinetics as in non-equilibrium gating[19], and or via increased late channel current[12], [20], will mask outward current during repolarization and exacerbate mutation-induced action potential prolongation. Figure 7 summarizes experiments designed to detect whether or not the F1473C mutation augments inward Na^+^ channel current under conditions that are similar to repolarization of the ventricle, and further, whether or not mexiletine and ranolazine are effective inhibitors of this current using previously-described negative ramp voltage protocols (Methods)[19]. Illustrated in the figure, are results of experiments in which cells were first depolarized to +20 mV for 100 ms to promote open state inactivation, after which repolarization to −100 mV was imposed with a negative voltage ramp, roughly mimicking the repolarization phase of the ventricular action potential. Figure 7A compares the response of WT channels with F1473C channels to this voltage protocol and several consequences of the mutation are readily apparent. First, compared with WT channels there is a very large increase in non-inactivating current for F1743C channels over the duration of the voltage pulse to +20 mV, confirming that the mutation-induced late current is evident beyond the range of inactivation/activation curve overlap (window current). Second, and importantly, the mutation induces a large surge of inward current during the negative repolarizing voltage ramp that will contribute greatly to delayed repolarization of myocytes expressing this channel (see discussion of model results). Mexiletine (Figure 7B) and ranolazine (Figure 7C) both markedly inhibit the mutation-induced inward current during the simulated plateau (100 ms at +20 mV) and repolarization phase (negative ramp) in this protocol. Thus, at concentrations with minimal effects on peak Na^+^ channel currents, both of these drugs are effective at inhibiting the deleterious functional effects of the F1473C mutation.

### Computational modeling: dramatic effects of the F1473C mutation on ventricular action potential duration and restoration of wild type cellular phenotype by Na^+^ channel blockers

In order to better understand the cellular phenotype caused by the mutation, the altered channel biophysics were incorporated into a Markov model of Na^+^ channel gating[21] and then inserted into an adult human ventricular action potential model[22], lacking a comparable model for ventricular cells in newborns. The sustained current observed in patch clamp experiments was modeled as an increased probability of entering a bursting mode of channel openings, based on the observed broad voltage-dependence of mutation-enhanced late current. The steady state inactivation shift was reproduced by altering the voltage dependence of the transition between closed and closed inactivated states which interestingly also recapitulated the observed speeding of recovery from inactivation determined in experiments (Figure 4B and Methods S1, Figure S-1).

**Figure 8:**
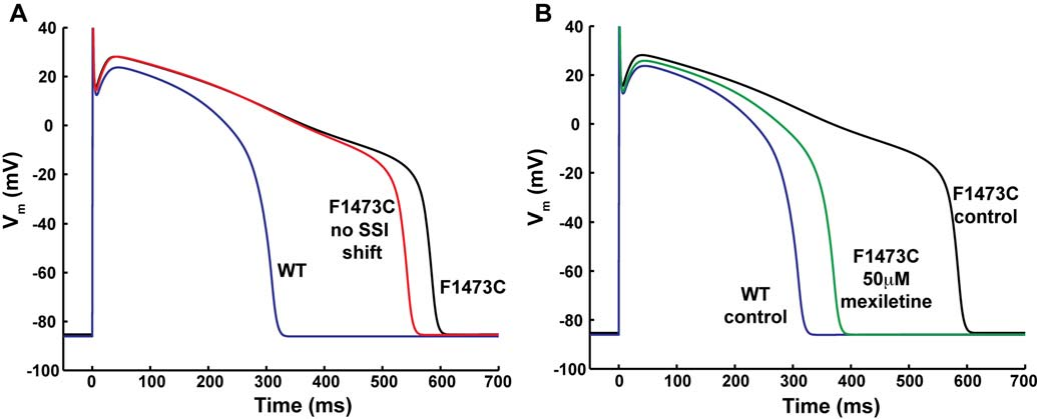
Simulation of the impact of the F1473C mutation and its modulation by mexiletine on the ventricular action potential. A. Shown are the computed action potentials for the F1473C mutant (black trace) as compared to WT (blue trace) at 1Hz. Also shown is the contribution of F1473C late current alone to action potential prolongation, without changing the channel steady state availability (red trace). B. The simulations show the drug markedly rescues the channel function reducing the F1473C APD from 588 ms to 386 ms.

Inserting the channel kinetics into a human ventricular action potential model results in a profound prolongation of the action potential. At a 1Hz pacing rate, steady state action potential duration was nearly doubled (313 ms, WT vs. 588 ms, F1473C) in the mutant (Figure 8A). Consistent with previously modeled LQT-3 mutants with a pronounced bursting mode, an increase in rate drastically reduced the APD difference between WT and mutant (at 2Hz, 252 ms WT, 344 ms F1473C). To dissect the individual contributions of the voltage shift in steady state inactivation (SSI) and the increase in bursting, each effect was simulated independently (at 1Hz). Interestingly the SSI shift alone had no effect on APD in the absence of bursting but in the presence of bursting its ablation abbreviated the action potential by 42 ms to 546 ms suggesting a significant contribution of the shift in addition to the bursting in the extremely prolonged QT intervals observed. Figure 8B, illustrates the simulated effects of 50 µM mexiletine on myocytes expressing the mutant channel by superimposing computed action potentials in the absence and presence of mexiletine (using drug block profiles in Table 1) for cells paced at 1Hz. Also shown in the figure for comparison is the control WT action potential of Figure 8A. Clearly, in the simulation mexiletine greatly rescues the channel defect reducing the F1473C cellular APD from 588ms to 386ms at 1Hz, close to the computed APD of cells expressing WT channels.

## Discussion

### Na^+^ channel variants and inherited arrhythmias in infants

The results of our study clearly show that the *SCN5A* F1473C mutation, which was discovered as a *de novo* mutation in a newborn, clearly disrupts inactivation of Na_v_1.5 channels in manners which contribute to delayed repolarization in cardiac cells expressing this channel variant. The present study adds to a growing literature that suggests that LQTS mutations in general and LQT-3 mutations in particular, are both more prevalent and particularly problematic in hearts of infants compared with adults[9]–[11], [23]–[26]. In a systematic study of autopsied SIDS cases Ackerman et al[27] reported an incidence of *SCN5A* mutations of 2.1% and most recently, in a survey of SIDS victims involving 201 Norewegian cases, approximately half of the gene variants linked to LQTS were found to be *SCN5A* mutations[7], which is higher than the approximately 10% incidence reported in adult LQTS patients[28].

Several characteristics of the clinical phenotype and therapeutic responses we report are consistent with those previously described for LQT-3 cases in infants, including both pronounced QT prolongation (594 ms to 740 ms[9], [11], [26]) and mixed efficacy in the controlling QT prolongation and resulting arrhythmias with Na^+^ channel blockers[9], [11], [24], [26]. For example, whereas mexiletine was effective at controlling both 2∶1 atrioventricular (AV) block in a newborn with an *SCN5A* (P1332L) mutation[11], in a another case neither mexiletine nor flecainide was useful in long-term control of QT prolongation in a neonate with another *SCN5A* mutation (R1623Q)[9]. In a different neonate with the R1623Q *SCN5A* mutation, mexiletine was initially effective in controlling QT prolongation, but it was difficult to maintain a therapeutic concentration, possibly due to poor absorption from the gastrointestinal track[24]. The variable efficacy of Na^+^ channel blockers in infants with the R1623Q mutation is particularly important in view of the fact that cellular biophysical and pharmacological studies showed effective restoration of channel function of this mutation by lidocaine[29].

### F1473C: a *de novo* mutation with severe biophysical consequences

Our results indicate that, like many of the previously-reported *SCN5A* mutations in young children, the F1473C mutation, which appeared *de novo* in the proband of this study, causes marked changes in Na_v_1.5 channel inactivation that are predicted to have severe physiological consequences. The most obvious effect of the mutation is an increase in late Na^+^ channel current over a broad range of cellular membrane potentials, an effect similar to other previously-described LQT-3 mutations such as the ΔKPQ mutation[30]. In addition, the F1473C mutation speeds recovery from inactivation, causes a positive shift in the voltages over which closed-state inactivation transitions occur, and produces a positive shift in the voltage-range over which steady-state inactivation and activation overlap (window current). Each of these perturbations alone has been shown to be sufficient to account for delayed repolarization for other LQT-3 mutations[19], [31]. Thus, it is not surprising that the patient carrying the F1473C mutation presented with extreme QT prolongation (approximately 800 ms). The concomitant 2∶1 heart block was very likely caused by conduction block in the ventricle due to extremely prolonged ventricular action potentials, similar to the suggested mechanism of 2∶1 heart block caused by another *SCN5A* mutation in a newborn[24]. Thus, similar to the recent report of Wang et al[8], we find that the F1473C mutation disrupts Na^+^ channel inactivation with severe predicted cellular phenotypic consequences. However, our analysis also suggests that the F1473C mutation-induced changes in Na^+^ channel activity by themselves are not sufficient to explain either the patient's disease phenotype or response to the different Na^+^ channel blockers.

### Impact of ionic and genetic background on phenotype of F1473C carriers: insight from computational modeling

Incorporation of the F1473C mutation-induced biophysical changes in channel gating described above into a model of an adult human ventricular myocyte[22] yielded simulated action potentials with markedly delayed repolarization that are qualitatively similar to the patient's phenotype. Simulations suggest both an increase in pacing rate and Na^+^ channel blockers as beneficial in recovering channel function consistent with the successful arrhythmia management obtained by pacing and high-dose mexiletine. The simulations also suggest that the absence of an effect of flecainide on Na^+^ channel closed state availability contributes to the ineffectiveness of this drug on the treatment of the proband.

### Limitations of the analysis and implications for roles of age and genetic background

Despite these important insights there are limitations that must be considered. First, the computational model is based on adult cardiac electrophysiology, because, at this time, appropriate models have not been developed for infants. This very likely affects the background of repolarizing potassium channel currents against which the F1743C sodium channel is simulated[32], [33]. Differences in resting potentials that may arise as a consequence of age-related repression of potassium channels[34] would likely exacerbate differences in drug action that affect closed state inactivation, in comparing flecainide and mexiletine effects in the proband. Additionally, flecainide, but not mexiletine, has been reported to inhibit I_Kr_ potassium channels for which HERG forms the principal α subunit[35], [36]. This effect might be tolerated in treatment of adult LQT-3 mutation carriers but it might offset the effects of flecainide on F1473C mutant channel activity in the mutation carrier due to his age and genetic background. Finally, the genetic background of the newborn must be considered. The patient carrying the F1473C mutation was also found to carry a common polymorphism *KCNH2* (K897T) [37]–[40]. This polymorphism has been studied extensively and found to affect HERG channel function, but there is no clear consensus on whether the altered function favors QT prolongation, shortening, or a combination of the two depending on physiological conditions such as heart rate[39]–[42]. Importantly, the mother of the proband, homozygous for K897T *KCNH2* was unaffected (normal QTc). Thus, it is possible that alteration in repolarization reserve via K897T HERG channels, while insufficient to affect electrical activity in the mother, may contribute to QT prolongation and, again to distinction in flecainide's activity due to this drug's effects on the I_Kr_ channel. This is likely to be the case as the K897T *KCNH2* has been reported to be a genetic modifier of latent LQTS in a carrier of K897T *KCNH2* and a another low-penetrant *KCNH2* mutation[42].

### Conclusions

In summary, we report and present biophysical and pharmacological analysis of a novel de novo *SCN5A* mutation that was discovered in a newborn diagnosed with severe LQTS providing additional evidence of the importance of Na^+^ channel mutations in inherited arrhythmias in newborns. Further, the very pronounced QT prolongation in the proband and the distinct therapeutic effectiveness of different Na^+^ channel blockers used to treat the patient were in large part due to the Na^+^ channel dysfunction caused by the mutation. However, our analysis suggests that the patient's genetic background and age impact both the severity of the disease phenotype and the differences in the effectiveness of two sodium channel blockers, flecainide and mexiletine as therapeutic agents. Our results underscore that mutation-induced changes in channel gating are necessary but not sufficient to explain the phenotypic and therapeutic responsiveness of patients harboring specific gene mutations. While distinct biophysical changes in channel properties will account for some drug discrimination in patients [43] other factors, such as the presence of polymorphisms in other channels (as in our patient) and age-related changes in cell physiology, must also be taken into account.

## Materials and Methods

### Genetics

The proband was bi-directionally sequenced for all the coding exons of *KCNE1*, *KCNE2*, *KCNH2*, *KvLQT1*, and *SCN5A*. 100 normal Caucasian controls with normal QT intervals on EKG and without a clinical history of syncope, presyncope or arrhythmias were screened for the F1473C *SCN5A*.

### Electrophysiology

Site-directed mutagenesis was done on Na_V_1.5 in pcDNA3.1 using the Quik Change site-directed mutagenesis kit (Stratagene). Whole cell recordings were made on Human Embryonic Kidney (HEK) 293 cells expressing Wild type and mutant Na_V_1.5 channels along with hβ1 subunits (Lipofectamine, Invitrogen) as previously described [31]. Solutions and voltage protocols are detailed in the supplemental materials (Methods S1). Mexiletine and Flecainide were provided by Sigma (St Louis, Missouri). Ranolazine was provided by CVT Therapeutics (Palo Alto, CA). Membrane currents were measured with Axopatch 200B amplifiers (Axon Instruments, Foster City, CA). Capacitance and series resistance compensation were carried out using analog techniques according to the amplifier manufacturer (Axon Instruments, Foster City, CA). PClamp8 (Axon Instruments) was used for data acquisition and initial analysis. Analysis was carried out in Excel (Microsoft), Origin 7.0 (Microcal Software, Northampton, MA), and programs written in Matlab (*The Mathworks,* Natick, MA). Analyzed data are shown as mean +/− S.E.M. Statistical significance was determined in some cases using the unpaired Student's t test and in others using the paired Student's t test; p<0.05 was considered statistically significant.

### Computational Methods

Sodium channel kinetics were modeled in the context of a Markov model based on previous work of Clancy et al,[21] and described in detail in Methods S1. The Markov model is then inserted into a human ventricular action potential model[22] and paced with twice threshold stimuli at a variety of pacing intervals to examine the cellular consequence of the mutation.

## Supporting Information

Methods S1 Supplemental methodology.
(0.05 MB DOC)
