# Supplementary material for: A Novel and Lethal De Novo LQT-3 Mutation in a Newborn with Distinct Molecular Pharmacology and Therapeutic Response
Source: PLoS One. 2007 Dec 5;2(12):e1258. doi: 10.1371/journal.pone.0001258 (PMC2082660; doi:10.1371/journal.pone.0001258)
Supplement: Methods S1 — Supplemental methodology. (0.05 MB DOC) [file pone.0001258.s001.doc]

Methods

Genetics

Genomic DNA was isolated from whole blood by cell lysis followed by DNA extraction and precipitation according to manufacturer's instructions (Promega; Madison, WI) and stored at 4 ^{o}C. The proband was bi-directionally sequenced for all the coding exons of *KCNE1*, *KCNE2*, *KCNH2*, *KvLQT1*, *and SCN5A*. When sequence variants were identified, the parents were sequenced only for the exon containing the genetic variant. PCR reactions for amplification of the exons consisted of 20 ml reaction volumes comprised of 100 ng genomic DNA, 1X reaction buffer supplied by Boehringer Mannheim in which the [$MgCl_2$] was 1.5 mM, 0.25 mM each dNTP, 100 ng of each PCR primer, and 1 U Taq polymerase. Primer sequences for the mutation-containing exon were:

SCN5A F1473 F	TGGGTCTTTCTTCACCCTGA
SCN5A F1473 R	AGAAGAGGACCATCCCCAAC.

All thermocycling was performed with 35 cycles of denaturation at 94°C for 30 seconds, annealing at 58°C, for 30 seconds and extension at 72°C for 30 seconds. PCR products were purified after electrophoresis through a 2% agarose gel using the Qiaquick DNA purification columns (Qiagen Inc., Valencia, CA). Fluorescent dideoxy termination sequencing of purified PCR products was performed using an ABI 377 sequencer using standard reagents and conditions as recommended by the manufacturer. Sequence was analyzed using SEQUENCHER software to compare subjects' sequence to control Caucasians without long QT syndrome or arrhythmias and the published reference sequence. In addition, each electropherogram was visually reviewed to identify any heterozygous DNA variants not detected by the automated sequencing software.

The proband and parents were analyzed for maternity and paternity using a panel of 15 microsatellite markers used for identity testing according to manufacturer's instructions (Applied Biosystems: Foster City, CA).

100 normal Caucasian controls with normal QT intervals on ECG and without a clinical history of syncope, presyncope or arrhythmias were screened for the SCN5A F1473C mutation by pyrosequencing. Pyrosequencing was performed according to manufacturer's instructions (PSQ96 Biotage, LLC. Westborough, MA) using a vacuum system with streptavidin sepharose beads (Amersham Biosciences AB, Uppsala Sweden); PCR reactions consisted of 5 pmol of each of the appropriate forward and reverse primer (F: 5'TGGGTCTTTCTTCACCCTGA and R biotin labeled: 5'AGAAGAGGACCATCCCCAAC), 1 U AccuPrime GC-Rich DNA polymerase (Invitrogen), 1x buffer A, and 30 ng of genomic DNA in a 25 ml reaction volume for 30 cycles at an annealing temperature of 55°C. The sequencing primer 5'TGGTGTCATCATTGACAACT was used to sequence the 8 base pairs encompassing the polymorphic site.

Electrophysiology

Patch clamp procedures were used in the whole cell recording mode with the following internal solution (in mM): 50 aspartic acid, 60 CsCl, 5 Na_2ATP, 11 EGTA, 10 HEPES, 4.27 $CaCl_2$ (resulting in a final $[Ca^{2+}]_i$ of 100nM), and 1 $MgCl_2$, pH 7.4 adjusted with CsOH. The external solutions for measurement of all Na^+ channel activity except activation curves contained (in mM): 130 NaCl, 2 $CaCl_2$, 5 CsCl, 1.2 $MgCl_2$, 10 HEPES, and 5 glucose, pH 7.4 adjusted with NaOH. In experiments designed to measure the voltage dependence of activation and the onset of inactivation, external Na^+ was

reduced to 30mM using n-methyl-glucamine as a Na^+ substitute to ensure adequate voltage control. The voltage dependence of inactivation was determined by measuring current at -10 mV after application of conditioning pulses (-120 mV to -20mV, 500 ms or 5s as indicated in figure captions) applied once every 15 sec. Currents were normalized to currents measured after the -120 mV conditioning pulse. To measure recovery from inactivation, conditioning voltage pulses (50 ms pulses from –100 mV to –10 mV) were followed by test pulses (- 10 mV, 10 ms) applied after a series of recovery times at -100 mV. Late non-inactivated sodium current (I_{NaL}) was measured as the tetrodotoxin (TTX; 50µM)-sensitive current measured at 200 ms during depolarization to -10 mV. I_{NaL} was normalized to peak TTX-sensitive Na^+ channel current measured at -10 mV and plotted as percentage of peak current in relevant figures. Positive voltage ramp protocols consisted of a voltage ramp applied from -120 mV to +40 mV at a rate of 0.16 V/sec. Negative voltage ramp protocols were used to study Na^+ channel current during repolarization. Here voltage pulses (100 msec), imposed to +20 mV to promote open state inactivation, were followed by negative voltage ramps (- 1.6 V/sec) to the holding potential. Unless otherwise specified, the holding potential was -100mV.

Computational Methods

The Markov model is that published previously [1]. Below are the rate constants used to reproduce the data from this study.

Wild-type rate constants

α11 = 3.802/(0.1027*exp(-v/17.0)+0.20*exp(-v/150));

α12 = 3.802/(0.1027*exp(-v/15.0)+0.23*exp(-v/150));

α13 = 3.802/(0.1027*exp(-v/12.0)+0.25*exp(-v/150));

$\beta 11 = 0.4*\exp(-v/20.3);$

$\beta 12 = 0.4*\exp(-(v-5)/20.3);$

$\beta 13 = 0.4*\exp(-(v-10)/20.3);$

$\alpha 3 = 1.897e-6*\exp(-(v+10)/7.7);$

$\beta 3 = (0.0084+.00002*(v));$

$\alpha 2 = (9.178*\exp(v/29.68))/2.5;$

$\beta 2 = ((\alpha 13*\alpha 3*\alpha 2)/((\beta 13*\beta 3)));$

$\alpha 4 = (\alpha 2/100)*1.5;$

$\beta 4 = \alpha 3*5;$

$\alpha 5 = (\alpha 2/95000)*1.5;$

$\beta 5 = (\alpha 3*5)/50;$

$\mu 1=5.3e-8;$

$\mu 2=3.0e-4;$

F1473C modifications

$\alpha 3 = 1.897e-6*\exp(-v/7.7);$

$\mu 1=1.0e-6;$

$\mu 2=3.0e-4;$

Changes in rate constants reproduce experimental observed kinetic changes.

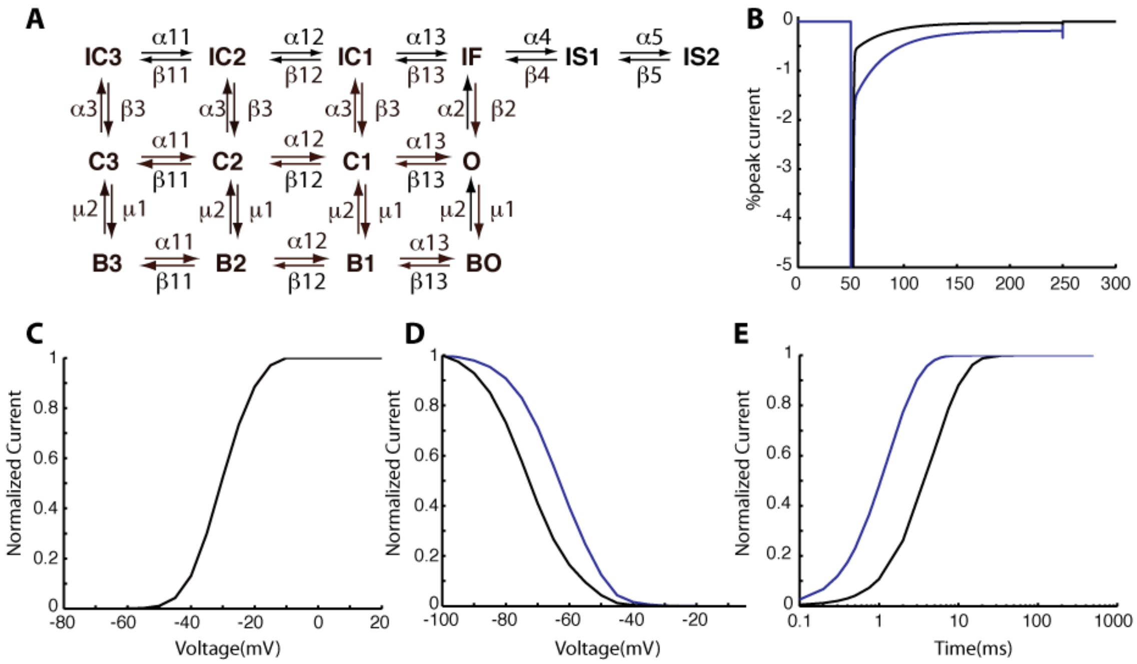

Figure S-1: Markov model of mutant channels reproduced the increase in late current (panel A), depolarizing shift in stead-state inactivation (panel C), and shift in recovery kinetics (panel E) seen experimentally. In all panels black lines are wild-type channels and blue lines are F1473C channels.

Literature Cited:

1. Clancy CE, Zhu ZI, Rudy Y (2007) Pharmacogenetics and anti-arrhythmic drug therapy: a theoretical investigation. Am J Physiol Heart Circ Physiol 292: H66-75.
